# A novel method to detect articular chondrocyte death during early stages of osteoarthritis using a non-invasive ApoPep-1 probe

**DOI:** 10.1186/s13075-015-0832-x

**Published:** 2015-11-04

**Authors:** Xiangguo Che, Lianhua Chi, Clara Yongjoo Park, Gyoung-Ho Cho, Narae Park, Seong-Gon Kim, Byung-Heon Lee, Je-Yong Choi

**Affiliations:** Department of Biochemistry and Cell Biology, Kyungpook National University School of Medicine, Daegu, 700-422 Republic of Korea; Department of Oral and Maxillofacial Surgery, College of Dentistry, Gangneung-Wonju National University, Gangneung, 210-702 Republic of Korea

**Keywords:** Early diagnosis of osteoarthritis, Articular chondrocyte, Apoptosis, ApoPep-1

## Abstract

**Introduction:**

Current methods for early diagnosis of osteoarthritis (OA) are limited. We assessed whether in vivo detection of chondrocyte death by ApoPep-1 (CQRPPR), a peptide that binds to histone H1 of apoptotic and necrotic cells, could be used to detect the initiation of OA.

**Methods:**

Apoptosis-induced ATDC5 cells were labeled with Annexin V and ApoPep-1. Surgical destabilization of the medial meniscus (DMM) was performed on both knees of 12-week-old male mice and severity of OA was determined by histological analysis according to the Osteoarthritis Research Society International (OARSI) guidelines. At 1, 2, 4, and 8 weeks post-surgery, mice were intravenously injected with fluorescence-labeled ApoPep-1 or control peptide and in vivo imaging was performed within 30 minutes of injection by near-infrared fluorescence (NIRF). Binding of ApoPep-1 to OA joints was demonstrated by ex vivo imaging and immunofluorescent staining using TUNEL and histone H1 and type II collagen antibodies.

**Results:**

Strong signals of ApoPep-1 were observed on the apoptotic ATDC5 cells. Knees corresponded to grade II, III, and V OA at 2, 4, and 8 weeks after DMM, respectively. Between 2 and 8 weeks after surgery, the in vivo NIRF signal at OA-ApoPep1-injected joints was consistently stronger than sham-operated or OA-control peptide-injected joints. ApoPep-1, TUNEL, and histone H1 signals were stronger in grade II OA cartilage than sham-operated cartilage when detected by immunofluorescent staining. Type II collagen expression was similar between grade II OA and sham group.

**Conclusion:**

ApoPep-1 can be used to detect OA in vivo by binding to apoptotic chondrocytes. This is a novel, sensitive, and rapid method which can detect apoptotic cells in OA rodent models soon after its onset.

## Introduction

Osteoarthritis (OA), which affects more than 46.4 million people in the USA, is characterized by gradual loss of cartilage [1–3]. The current available treatment for OA is joint replacement therapy, which is performed when the cartilage is severely damaged [4]. This late-stage OA is preceded by chronic pain and difficulty in mobility [5, 6]. It is thought that non-surgical treatment of OA is only possible during its early development, but early detection of the disease is difficult due to the lack of symptoms and affordable and sensitive detection methods. Until further medical treatment techniques are developed, diagnosis soon after the onset of OA is essential to prevent irreversible cartilage degeneration and deterioration and to promote cartilage recovery.

Current OA diagnosis methods have limitations for the early detection during the onset of OA. The sensitivity of radiography is insufficient to detect the onset of OA. Radiography can only examine joint space narrowing and osteophytes, which are characteristics of advanced OA [7]. Magnetic resonance imaging (MRI) provides high-resolution computerized images of internal body tissue, but is limited in its ability to detect articular chondrocyte apoptosis, proteoglycans, and aggrecan loss, which are characteristics of the initiation of OA. Loss of type II collagen, an event that occurs in the early stages of OA development [8, 9], is not detectable by MRI. Techniques for diagnosing the onset of OA are necessary to prevent chronic pain, prolonged difficulty in mobility, possible complications due to joint replacement surgery, and increased healthcare costs for the patient and society [10, 11].

Detecting the amount of apoptotic articular chondrocytes may be a useful approach to diagnosis of OA, because apoptosis occurs at an increased rate in the cartilage in OA compared to healthy cartilage [12, 13]. Articular chondrocytes are important in maintaining the dynamic equilibrium between synthesis and degradation of the extracellular matrix [12–14]. Although subchondral bone change has been postulated to be the first sign of initiation of OA [15], articular change is also one of the first to be affected by OA and its destruction can be a warning sign of OA progression [15–19]. During the early stages of OA, chondrocyte apoptosis increases in the articular surface and middle zones of the cartilage, probably as a consequence of constant mechanical damage to the joint [20–22]. Apoptotic chondrocyte death [23] has been reported to occur more frequently in osteoarthritic cartilage than healthy cartilage in humans and animals, and has been positively correlated with the severity of cartilage damage in the joint [22, 24–26]. Apoptosis indicators include exposed cellular phosphatidylserine, dysfunctional mitochondria, activated caspases, fragmented DNA, and disrupted membrane integrity [27–29]. Annexin V, a 36 kDa protein that binds to phosphatidylserine, is most commonly used and generally considered an early marker of apoptotic cell death in vitro. However, annexin V also binds to type II collagen, which is highly expressed in healthy articular cartilage and facilitates the binding of chondrocytes to collagen [30–33]. Therefore, annexin V would not be suitable for distinguishing osteoarthritc cartilage from healthy cartilage. On the other hand, caspase antibodies bind to caspase enzymes to detect programmed cell death, but these antibodies have slow binding kinetics, delayed clearance, and the possibility of immunogenicity. These drawbacks require the development of a new in vivo method to diagnose OA during its initial stages.

Peptides have attractive potential to be used in diagnostic tools because of their many advantages, including rapid binding kinetics and degradation, minimal concern for immunogenicity, structural diversity, and attachability to diverse probes [34–37]. Therefore, when used for the diagnosis of OA, peptides may safely and accurately detect osteoarthritic cartilage within a shorter time span and be rapidly excreted from or degraded in the body. Apoptosis-targeting Peptide-1 (ApoPep-1), a six-amino-acid CQRPPR peptide, recognizes apoptotic and necrotic cells by binding to histone H1 exposed on the surface of these cells [38]. Histone H1, a linker histone which helps stabilize chromatin fiber, is abundant in the nucleus but is exposed on the cell membrane and released into the extracellular matrix during apoptosis, thereby serving as a unique and specific marker for cell death [39–41]. Use of ApoPep-1 conjugated with fluorescent dye or isotopes has been successful for in vivo imaging of apoptosis in tumor cells and neurons [38, 42, 43].

In this study, we examined whether ApoPep-1 is effective in detecting OA during its early stage in vivo. Destabilization of the medial meniscus (DMM) [44] was performed on male mice and signals were assessed 2–8 weeks post-surgery.

## Methods

### Cell culture and immunocytochemistry

ATDC5 cells were maintained in a mixture of DMEM and Ham’s F-12 medium (DMEM/F12) (Lonza, Walkersville, MD, USA) containing 5 % fetal bovine serum (FBS; Gibco-BRL, USA), 10 g/ml of human transferrin (Sigma, St. Louis, MO, USA), 3 × 10^8^ M of sodium selenite (Sigma), and penicillin/streptomycin (Lonza). ATDC5 cells were plated at a density of 1 × 10^5^ cells/ml and apoptosis was induced by incubating cells with 0.5 mM staurosporine (Sigma) for 6 hours. Cells were stained with annexin V (Santacruz, Dallas, TX, USA) and FlammaTM 675 conjugated ApoPep-1 (BioActs, Namdong-gu, Incheon, Korea) to determine cell apoptosis. For immunocytochemistry, cells were blocked in 1 % bovine serum albumin (BSA) at 37 °C for 30 minutes and then for cell internalization samples were soaked in 10 μM FlammaTM 675-conjugated peptide at 4 °C for 1 hour. Cells were then co-stained with Alexa-594-annexin V (Invitrogen, Carlsbad, CA, USA) for 15 minutes at room temperature (RT) in binding buffer (10 mM HEPES, pH 7.4, 140 mM NaCl, and 2.5 mM CaCl_2_). After fixation, cells were incubated with 4′, 6′ -diamidino-2-phenylindole (DAPI, Sigma-Aldrich) to stain the nuclei. Photos of the stained cells and differential interference contrast (DIC) images were taken using a Zeiss LSM-510 Meta confocal microscope (Zeiss, Oberkochen, Germany) using a plan apochromat 20×/0.8 numerical aperture.

### Experimental animals

**Fig. 1:**
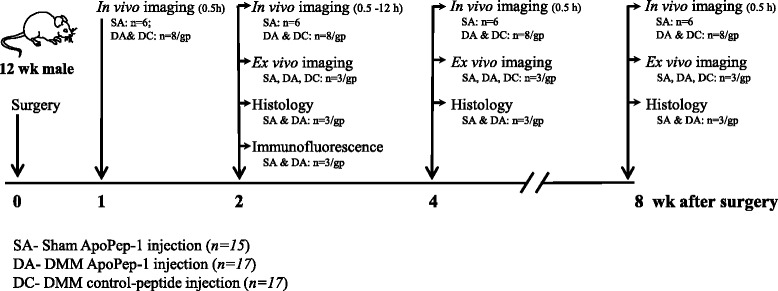
Experimental scheme. Osteoarthritis was induced by surgical destabilization of the medial meniscus (*DMM*). Mice were divided into four experimental groups: one that underwent in vivo imaging (n = 22) and three other groups that underwent ex vivo imaging, histology and immunofluorescence analysis (n = 27). *SA* sham ApoPep-1 injection, *DA* DMM ApoPep-1 injection, *DC* DMM control-peptide injection, *gp* group

C57BL/6 N mice were purchased from KOATECH (Gyeonggi-do, Korea), and animal care and experiments were carried out in accordance with the Institutional Animal Care and Use Committee of Kyungpook National University (KNU 2011-68). Animals were maintained on a 12-hour light/12-hour dark cycle at 22–25 °C in a specific pathogen-free environment and fed standard rodent chow and water *ad libitum*. Destabilization of the medial meniscus (DMM) surgery [44, 45] or sham surgery was performed in a total of 49 male mice at 12 weeks of age. The experimental scheme is shown in Fig. 1.

### Assessment of OA

Three mice were sacrificed per time point at 2, 4, and 8 weeks after DMM surgery to assess the degree of OA. An additional three mice were used as sham controls, which were sacrificed 2 weeks after sham surgery. Hind limbs of mice were fixed with 4 % paraformaldehyde (PFA) in 0.1 M PBS for 12 hours and decalcified with 10 % ethylenediaminetetraacetic acid (EDTA; pH 7.4) for 3 weeks. Decalcified tissues were dehydrated by soaking the samples in increasing concentrations of ethanol and embedding in paraffin, and then were sectioned into a thickness of 3 μm. For Safranin-O staining, sections were deparaffinized, rehydrated, dipped in Weigert’s iron hematoxylin (Sigma-Aldrich) for 10 minutes, in fast green solution (Sigma) for 5 minutes, and in 0.1 % Safranin-O solution (Sigma) for 5 minutes. Joints were assessed for severity of OA according to the Osteoarthritis Research Society International (OARSI) diagnosis criteria [46]. Briefly, grade I and II denote that the superficial zone remains intact, although there may be some microscopic fibrillation and fissuring, and the middle and deep zones are unaffected. Grade III changes appear when vertical fissures extend into the middle zone, but there is still no significant cartilage loss. Grade IV OA develops when increased fissuring results in cartilage erosion. Grade V and VI OA describe almost complete erosion of the articular cartilage with changes affecting the underlying bone, such as sclerosis [47].

### In vivo and ex vivo optical imaging

Among the mice that underwent surgical DMM, half were injected with FlammaTM675-ApoPep-1 (Bioacts) and the other half with FlammaTM675-NSSSVDK (control peptide; Bioacts) through the tail vein (100 nmol/20 g body weight) (n = 8/group). FlammaTM675-ApoPep-1 solution (1 mM) was diluted with PBS before the injection. Sham-operated mice were also injected with FlammaTM675-ApoPep-1 (n = 6). In vivo optical imaging was performed 0.5, 2.0, 4.0 and 12.0 hours after probe administration using eXplore Optix (ART, Montreal, QC, Canada) at 2 weeks after surgery, and at 0.5 hours after injection at 1, 2, 4, and 8 weeks after surgery. eXplore Optix measures fluorescent photons following excitation by a picoseconds laser (laser pulse: 80 MHz). Mice were put under anesthesia by inhalation of isoflurane (JW Pharmaceutical, Seoul, Korea) in 80 % N_2_O/20 % O_2_ in the supine position and the field of view for optical imaging was set manually in the joint or abdomen region of the mice. The excitation/emission wavelengths for FlammaTM675 were 676 nm/704 nm. Ex vivo optical imaging was performed in extracted hind limbs using the same method (n = 3/group).

### Immunofluorescence staining

At 2 weeks post-operation (grade II OA), three mice per group were sacrificed and knee joints were collected two hours after injection of FlammaTM675-ApoPep1. The joints were fixed with 4 % PFA in 0.1 M PBS for 12 hours and decalcified with 10 % EDTA (pH 7.4) for 3 weeks. Samples were protected from light with aluminum foil. Decalcified tissues were soaked in 20 % sucrose/PBS solution overnight and embed in optimal cutting temperature compound (OCT) (Tissue-Tek, Torrance, CA, USA) and stored at –80 °C. Frozen samples were cut at 10 μm thickness from the femur to the tibia to visualize the joint. Sections were washed with PBS, treated with blocking solution (0.1 % tween-20, 1 % BSA), 5 % normal donkey serum in PBS (Abcam, Cambridge, MA, USA)), and then incubated at 4 °C for 1 hour with primary antibodies (rabbit polyclonal collagen II antibody (1:200; Abcam), rabbit polyclonal histone H1.2 (1:200; Santa Cruz, CA, USA) and rabbit IgG (1:200; Abcam)). Sections were incubated with Alexa-594- and Alexa-488-conjugated secondary antibody for collagen type II and histone H1.2, respectively, for 40 minutes. Thereafter, the sections were incubated with DAPI for nuclei staining. Photos of the stained sections were taken using a Zeiss LSM-510 Meta confocal microscope (Zeiss) using a plan apochromat 10×/0.45 numerical aperture.

### TUNEL assay

TUNEL assay was conducted in the same joint samples that were used for immunofluorescence. The ApopTag® Red In Situ Apoptosis Detection Kit (Millipore, Billerica, MA, USA) was used according to the manufacturer’s instructions. Frozen sections were air-dried for 30 minutes at RT and washed three times with PBS for 3 minutes each. The sections were pre-fixed with 1 % PFA for 10 minutes at RT, and then post-fixed by pre-cooled ethanol/acetic acid (2:1) at –20 °C for 5 minutes. The fixed tissue sections were incubated with DAPI for nuclei staining following exposure to equilibration buffer for 10 seconds at RT and TdT enzymes for 30 minutes at 37 °C. Photos of the stained sections were taken using a Zeiss LSM-510 Meta confocal microscope (Zeiss).

### Statistics

Statistical analyses were performed using Sigma Plot software 10.0 (Systat Software Inc, Chicago, IL, USA). Data are expressed as mean ± SD. Differences between groups means were assessed by Student’s *t* test. *P* <0.05 was considered statistically significant.

## Results

### ApoPep-1 binds to apoptotic chondrocytes in vitro

**Fig. 2:**
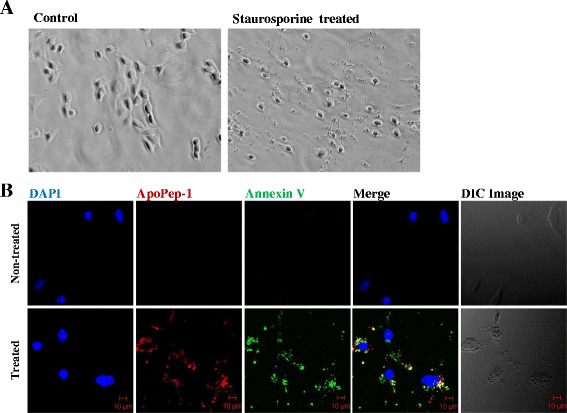
ApoPep-1 binds to apoptotic chondrocytes. **a** ATDC5 cells were incubated with staurosporine (0.5 μM) for 6 hours to induce apoptosis **b** ApoPep-1 binds to cells that bind to annexin V, a widely used in vitro marker for apoptosis. Cells were stained with ApoPep-1 (red), annexin V (green), and 4′, 6′ -diamidino-2-phenylindole (*DAPI*) (blue). Differential interference contrast (*DIC*) microscopy. Magnification, ×400

To define the binding ability of ApoPep-1 to apoptotic chondrocytes in vitro, we treated the ATDC5 chondrocyte cell line with staurosporine, which induces cell apoptosis [48, 49]. In vitro fluorescence revealed that strong signals of ApoPep-1 were observed on the cell membrane, extracellular and cytosol of apoptotic cells, but not on live cells (Fig. 2). Approximately 89 % of the cells bound with annexin V were also bound to ApoPep-1. However, ApoPep-1 and annexin V were not co-localized at the same spot on the apoptotic cells because ApoPep-1 binds to histone H1.2, whereas annexin V binds to phosphatidylserine, respectively. In these results we demonstrated that ApoPep-1 detects apoptotic chondrocytes.

### ApoPep-1 detects early stage OA *in vivo*

**Fig. 3:**
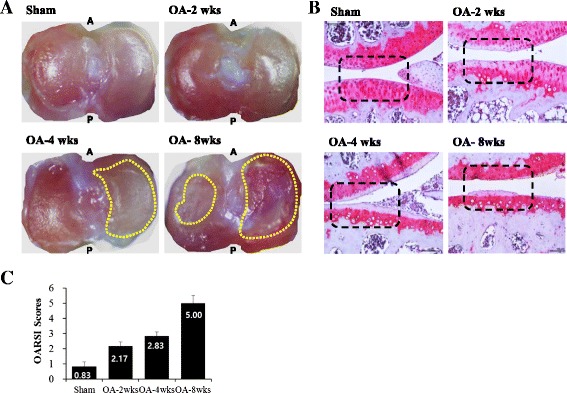
Morphologic and histology analyses during osteoarthritis (OA) progression. **a** Morphology of the left proximal tibial cartilage of mice that received sham operation or surgical destabilization of the medial meniscus (DMM). Eroded surfaces are seen within the *yellow dotted lines*. **b** Histomorphometry of joints in sham-operated and mice that underwent DMM, shown by Safranin O staining. **c** Mean Osteoarthritis Research Society International (*OARSI*) scores for joints for indicated surgery/time points (n = 3/group). Values are mean ± SD. *A* anterior, *P* posterior, *wks* weeks

**Fig. 4:**
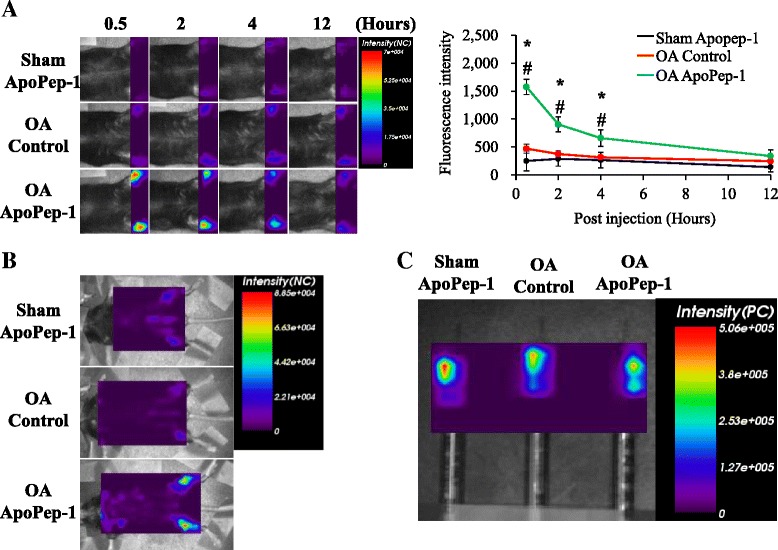
Osteoarthritis (*OA*) initiation is detected using ApoPep-1 within 30 minutes of injection. Destabilization of the medial meniscus or sham surgery was performed in both knees of the mice at 12 weeks of age and mice were scanned in the supine position 2 weeks after surgery. *Purple boxes* indicate region of scan. **a** ApoPep-1 was detected in joints of OA mice (2 weeks after surgery) from 0.5 hours to 4.0 hours after intravenous injection. Signals disappeared by 12 hours. Photo of one representative mouse for each group. Quantified mean ± SD is shown for each group (arbitrary units). **b** Fluorescence is detected in the joints of OA mice injected with ApoPep-1, but not in other organs, sham mice, or mice injected with the control peptide. Photo of one representative mouse for each group. *Sham-ApoPep-1* sham operation and Apo-Pep-1 injection (n = 6), *OA-Control* OA operation and control peptide injection (n = 8), *OA-ApoPep-1* OA operation and ApoPep-1 injection (n = 8), *NC* normalized count. Values are mean ± SD; *p <0.01 OA-ApoPep-1 compared to Sham-ApoPep-1, ^#^ *p* <0.01 OA-ApoPep-1 compared to OA-Control. **c** Fluorescence intensity (arbitrary units) of ApoPep-1 and control peptide were similar before injection. *PC* proton count

Given the in vitro results, we sought to determine whether ApoPep-1 could be used to detect initiation of OA using in vivo imaging. Sham-operated mice had healthy cartilage surfaces with clear boundaries between the cartilage and calcified bone surfaces 2 weeks post-surgery. The pink stain (Safranin O) is an indicator of proteoglycan, which is abundant in healthy cartilage. The intensity of pink staining is proportional to proteoglycan content. The decreased intensity of Safranin O with OA progression indicates loss of proteoglycan and thus, damage to the cartilage over time after DMM. In addition, the cartilage surface had severe degeneration 8 weeks post-surgery (Fig. 3a-b). However, joints of mice that underwent surgical DMM matched the description of grade II, III, and V OA at 2, 4, and 8 weeks post-surgery, respectively, according to the OARSI diagnosis criteria [46]. Diagnosis of OA is required at the latest by the time grade III is reached, as OA up to grade III is thought to have higher treatment efficacy and to be potentially reversible [4, 50–53]. No signals were detected in vivo in sham-operated mice and OA-model mice one week after surgery. From 30 minutes to 12 hours post-injection, a time-dependent decrease in fluorescent signals was detected when injected with ApoPep-1 at 2 weeks after DMM (grade II OA joints), but not in sham- or control-peptide-injected DMM joints despite the similar fluorescence signals of the peptides before injection (Fig. 4a-b). Signals completely disappeared by 12 hours in the OA-ApoPep-1 mice. The signals were specific to the joint and did not appear in other organs at 30 minutes after injection (Fig. 4c). These results indicate that ApoPep-1 specifically binds to joints with early OA within a short time period after injection, and that the peptide is rapidly excreted.

**Fig. 5:**
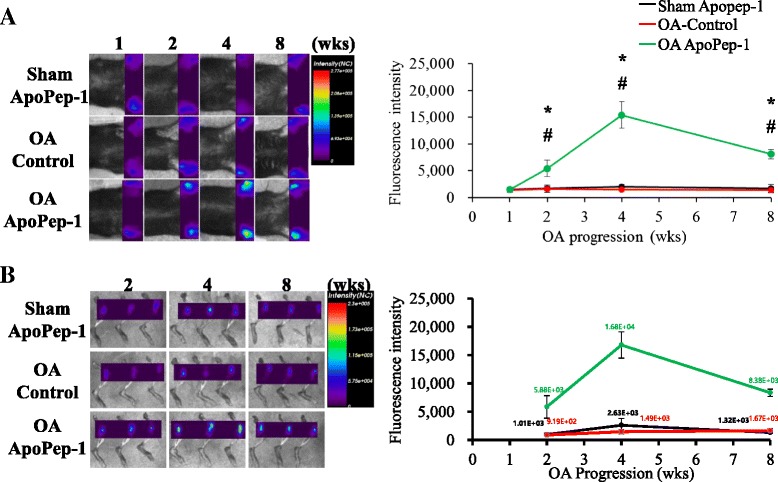
Assessment of osteoarthritis (*OA*) progression using the ApoPep-1 probe. **a** In vivo optical images 30 minutes after intravenous injection of ApoPep-1 or control probe at different stages of OA development (1, 2, 4, and 8 weeks after destabilization of the medial meniscus (DMM) or sham operation). Photo of one representative mouse for each group. Quantified mean ± SD are shown for each group (arbitrary units): *Sham-ApoPep-1* sham operation and Apo-Pep-1 injection (n = 6), *OA-Control* OA operation and control peptide injection (n = 8), *OA-ApoPep-1* OA operation and ApoPep-1 injection (n = 8), *wks* weeks. **b** Ex vivo optical images of mouse joints 2 hours after intravenous injection of ApoPep-1 at different stages of OA development (2, 4, and 8 weeks after sham operation or DMM) (n = 3/group). Quantified mean ± SD are shown for each group (arbitrary units); *p <0.05 OA-ApoPep-1 compared to Sham-ApoPep-1, ^#^ *p* <0.05 OA-ApoPep-1 compared to OA-Control. *NC* normalized count

To monitor the progression of OA with ApoPep-1, we assessed the strength of fluorescence at 1, 2, 4, and 8 weeks after surgery. Significantly stronger fluorescent signals were observed in OA joints at 2 weeks post-surgery and reached a peak at 4 weeks post-surgery in the in vivo samples (Fig. 5a). The reduced signal strength at 8 weeks post-DMM may be due to the absolute decrease in apoptotic chondrocytes following articular cartilage loss. Ex vivo near-infrared fluorescence (NIRF) imaging of joints was performed after FlammaTM675-ApoPep-1 injection also showed that the signals accumulated in the joints of OA mice at 2 weeks post-surgery and reached a peak at 4 weeks post-surgery, and that there was reduced signal strength at 8 weeks post-DMM (Fig. 5b). These results indicate that ApoPep-1 is a useful imaging probe to detect early-stage and later-stage OA, though signal strength may not correlate with OA damage.

### In situ assessment of ApoPep-1 in joints

**Fig. 6:**
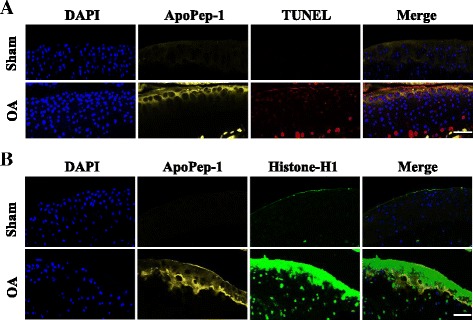
ApoPep-1 binds to grade II osteoarthritis (*OA*) cartilage. Joints were harvested and cryo-sectioned 2 weeks after sham operation or surgical destabilization of the medial meniscus. ApoPep-1 was injected intravenously before harvest. **a** Strong signals of ApoPep-1 and TUNEL both appear in grade II OA joints, but not in sham-operated joints. **b** ApoPep-1 and histone H1 appear in OA cartilage, but not sham-operated cartilage. *Scale bar* 50 μm. *DAPI* 4′, 6′ -diamidino-2-phenylindole

**Fig. 7:**
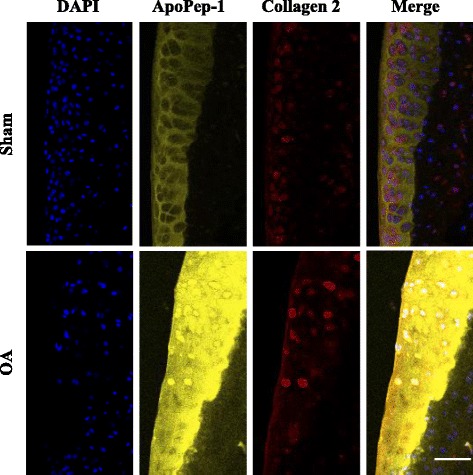
ApoPep-1 binds to grade II osteoarthritis (OA) cartilage before loss of type II collagen. ApoPep-1 was injected 2 weeks after sham operation or surgical destabilization of medial meniscus (DMM) and joints were harvested. The joints were cryo-sectioned and stained for 4′, 6′ -diamidino-2-phenylindole (*DAPI*, *blue*) and Collagen 2 (*red*). *Yellow* ApoPep-1. *Scale bar* 50 μm

Grade II OA joints were analyzed by in situ immunofluorescence staining to confirm the correlation of ApoPep-1 and cell death in cartilage at 2 weeks post-operation. Through immunofluorescence, strong signals of both ApoPep-1 and TUNEL were observed on the cell surface and extracellular matrix of OA joints (Fig. 6a). No signal was detected in sham articular cartilage. Fluorescence from FlammaTM675-ApoPep-1 was observed in OA joints at locations similar to but also deeper in the articular cartilage than histone H1 (Fig. 5b). ApoPep-1 was specifically accumulated in the articular cartilage but not in the subchondral bone or synovium tissues. Type II collagen expression was similar between the sham and OA mice articular cartilage, whereas strong ApoPep-1 signals were detected in the OA cartilage, indicating that ApoPep-1 can bind to cartilage before loss of type II collagen (Fig. 7). These in situ results suggest that ApoPep-1 can detect apoptotic chondrocytes through binding to histone H1 at early stages of OA, before loss of type II collagen is detected.

## Discussion

In this study, we found that the ApoPep-1 probe clearly detected surgically induced OA from its initiation (grade II) up to late-stage OA (grade V) on in vivo imaging. An ideal method should detect OA as early as possible, be rapidly accumulated in high concentrations in knee joints, and rapidly cleared from the system [47]. ApoPep-1 binds to grade II OA joints before loss of type II collagen, but not to healthy joints, and as early as 30 minutes post-injection. We observed higher type II collagen signals in the grade II OA cartilage compared to sham-operated cartilage.

In humans, chondrocytes in OA cartilage are reported to form clusters, which increase in size with OA progression [54]. These events may lead to aggregation of type II collagen in the cartilage and result in the stronger signals of type II collagen seen in our mice, confirming the initiation of OA. We did not find any signals in other organs, although other organs, such as the intestine, have rapid cell turnover rate and a large amount of apoptosis occurs. As the fluorescence intensity was normalized to eliminate background and auto fluorescence, fluorescent signals from other organs were dampened or disappeared while the strong signals at the OA joints were clearly detected. ApoPep-1 has been previously reported to be cleared from the system through the kidney within 4 hours of injection and did not accumulate in the liver and lung [38]. These results indicated that ApoPep-1 is optimal for use in vivo even when bound to materials with possible health risks following prolonged exposure, such as radioisotopes.

The rapid and highly concentrated binding of ApoPep-1 to OA joints shows that ApoPep-1 is an effective tool to detect OA during the early stages. The binding site of ApoPep-1 differs from that of annexin V and TUNEL in the apoptotic cells and OA tissues, respectively. Although both ApoPep-1 and annexin V were present on the surface of apoptotic ATDC5 cells in vitro, the specific binding sites did not always coincide. ApoPep-1 binds to histone H1.2, whereas annexin V binds to phosphatidylserine. Similarly, TUNEL staining, which detects fragmented DNA, appeared within the cell nucleus of the tissue, whereas ApoPep-1 was found on the cell surface and within the articular cartilage matrix. Histone H1 has been reported to be released into the extracellular matrix during apoptosis [39–41, 55]. On the other hand, the binding sites of histone H1 antibody and ApoPep-1 signals were similar in the articular cartilage but ApoPep-1 penetrated deep into the cartilage matrix despite being injected in vivo before preparation for immunofluorescence. ApoPep-1 may bind to other factors within OA cartilage once bound to damaged cartilage, but this still needs to be elucidated. Nonetheless, ApoPep-1, annexin V, TUNEL, and histone H1 antibody all bound to grade II OA joints, but not sham-operated joints.

ApoPep-1 has many strengths for its use in vivo. Recently, enhanced T1ρ-weighted MRI has been reported to measure biochemical composition and changes in proteoglycan content in articular cartilage during the initiation of OA [56]. However, MRI scanners are very expensive for general use (especially for the preventative use in those without symptoms), noisy, and may cause claustrophobia during the scan. MRI scans are also inappropriate for patients with cardiac pacemakers or other metal implants, which are not uncommon in the older population. The use of ApoPep-1 to detect OA is more affordable and easily generalizable than MRI. Moreover, ApoPep-1 can be easily synthesized and optimized, has no serious immune responses, and is rapidly cleared from the system. ApoPep-1 is non-toxic and does not accumulate in specific organs, which can minimize possible side effects of markers conjugated to ApoPep-1 (such as fluorescent dyes, radioactive isotopes, etc). The use of fluorescent probes conjugated with ApoPep-1 may be limited for detection of OA in humans. The eXplore Optix detects nanomolar concentrations of fluorescent signals at depths of 5–8 mm in mouse tissue. Thus, the thicker depth of skin and muscle in humans will hinder the detection of fluorescence signals transmitted from the ApoPep-1 probe in OA joints. For use in humans, ApoPep-1 may be conjugated with radioisotopes, while fluorescent probes may be more economical for in vivo OA research using surgically induced OA rodent models. In the future, ApoPep-1 conjugated with anti-apoptotic drugs may be used for targeted OA drug delivery.

One limitation of our study is that we did not test whether ApoPep-1 distinguishes OA from other joint diseases, such as rheumatoid arthritis (RA). However, the clinical symptoms differ between RA and OA, and do not necessarily need to be differentiated from each other by in vivo imaging. In addition, ApoPep-1 may not be used to monitor OA progression or treatment effects. Though OA joints had higher fluorescent signals than sham-operated joints, the degree of OA did not correlate with the strength of fluorescence, especially during later stages of OA, possibly due to the absolute loss of chondrocytes in advanced OA. However, the loss of chondrocytes and cartilage at this stage can be detected by MRI. The use of ApoPep-1 in humans must first be evaluated, as apoptosis occurs slowly in natural OA in humans. Acute conditions of high apoptosis following exercise or other stress-related conditions may lead to the misdiagnosis of OA. Therefore, for future application in clinical settings, patients will need to be counseled several days before the use of ApoPep-1. Still, our results indicate that ApoPep-1 is suitable for in vivo OA research during initiation of the disease, when using surgically induced OA rodent models.

## Conclusion

The ApoPep-1 probe facilitates the detection of OA as early as 2 weeks after DMM in mouse models (grade II OA) by specifically detecting apoptotic chondrocytes within 30 minutes. The present study is the first to detect initiation of OA in vivo during its potentially reversible period. ApoPep-1 is a potentially safe, effective, cost-effective and time-efficient method for the early detection of OA in rodent models.

## Abbreviations

BSA: Bovine serum albumin
CT: Computed tomography
DAPI: 4′, 6′-Diamidino-2-phenylindole
DMEM: Dulbecco’s modified Eagle’s medium
DMM: Destabilization of the medial meniscus
EDTA: Ethylenediaminetetraacetic acid
MRI: Magnetic resonance imaging
NIRF: Near-infrared fluorescence
OA: Osteoarthritis
OARSI: Osteoarthritis Research Society International
PBS: Phosphate-buffered saline
PFA: Paraformaldehyde
RT: Room temperature
